# Cholinergic Synaptic Transmissions Were Altered after Single Sevoflurane Exposure in *Drosophila* Pupa

**DOI:** 10.1155/2015/485709

**Published:** 2015-02-01

**Authors:** Rongfa Chen, Tao Zhang, Liting Kuang, Zhen Chen, Dongzhi Ran, Yang Niu, Kangqing Xu, Huaiyu Gu

**Affiliations:** ^1^Department of Anesthesiology, Peking University Shenzhen Hospital, Shenzhen 518000, China; ^2^Department of Anesthesiology, The First Affiliated Hospital of Sun Yat-Sen University, Guangzhou 510080, China; ^3^Department of Anatomy and Neurobiology, Zhongshan School of Medicine, Sun Yat-Sen University, Guangzhou 510080, China; ^4^Department of Rehabilitation, Shanghai First People's Hospital, Shanghai 201620, China

## Abstract

*Purpose*. Sevoflurane, one of the most used general anesthetics, is widely used in clinical practice all over the world. Previous studies indicated that sevoflurane could induce neuron apoptosis and neural deficit causing query in the safety of anesthesia using sevoflurane. The present study was designed to investigate the effects of sevoflurane on electrophysiology in *Drosophila* pupa whose excitatory neurotransmitter is acetylcholine early after sevoflurane exposure using whole brain recording technique. *Methods*. Wide types of *Drosophila* (canton-s flies) were allocated to control and sevoflurane groups randomly. Sevoflurane groups (1% sevoflurane; 2% sevoflurane; 3% sevoflurane) were exposed to sevoflurane and the exposure lasted 5 hours, respectively. All flies were subjected to electrophysiology experiment using patch clamp 24 hours after exposure. *Results*. The results showed that, 24 hours after sevoflurane exposure, frequency but not the amplitude of miniature excitatory postsynaptic currents (mEPSCs) was significantly reduced (P < 0.05). Furthermore, we explored the underlying mechanism and found that calcium currents density, which partially regulated the frequency of mEPSCs, was significantly reduced after sevoflurane exposure (P < 0.05). *Conclusions*. All these suggested that sevoflurane could alter the mEPSCs that are related to synaptic plasticity partially through modulating calcium channel early after sevoflurane exposure.

## 1. Introduction

Sevoflurane, an ether inhalational general anesthetic, with its low pungency, nonirritating odor, and low blood/gas partition coefficient, has been widely used in clinical anesthesia [1]. Additionally, since there is much advancement in surgery recently incidence and duration of exposure to sevoflurane in people receiving surgery are increasing. These raised concerns that whether anesthesia using sevoflurane would contribute to postoperative cognitive dysfunction (POCD) as rodent studies indicated that persistent learning deficit and social behavior dysfunction occurred after general anesthesia [2, 3]. Although recent clinical and laboratory data suggested that exposure to inhalational anesthetics is associated with subsequent learning and memory problems, mechanisms which underline these effects are still not clear. Previous studies indicated that isoflurane, thiopental, propofol, and ketamine would cause neurodegeneration and learning and memory deficiency [4, 5]. These suggested that the neurotoxicity induced by inhalational anesthetic might be mediated by activation of *γ*-aminobutyric acid (GABA) and inhibition of N-methyl-D-aspartic acid (NMDA) receptor [5–7].

Sevoflurane, like other inhalational anesthetic agents, activates *γ*-aminobutyric acid (GABA) and blocks N-methyl-D-aspartic acid (NMDA) receptor. Mounting data suggested that sevoflurane could produce histological damage in central nervous system (CNS) and subsequent alterations in behavior [8–10]. Meanwhile sevoflurane at clinical concentration could inhibit neurotransmission in a dose dependent manner in hippocampal slice study [11, 12]. Furthermore, a human volunteers study suggested that 0.25% sevoflurane blocks emotional memory [13]. The effects of sevoflurane on histological change and synaptic transmission of glutamatergic neurons have been well studied; however, there is little known about it on cholinergic neurons after sevoflurane exposure especially cholinergic synaptic transmission.

Cholinergic neurons function has been implicated in learning and memory processes [14]. Impairment of cholinergic system would cause disastrous loss of memory and learning ability [15, 16]. Therefore, it is possible that sevoflurane causes learning and memory impairment through damaging and disturbing cholinergic neurons function. However, cholinergic neurons are minority in mammal brain and it makes investigating cholinergic neurons function after sevoflurane exposure more difficulty.

*Drosophila melanogaster* has been widely used as model organism to study neuropharmacology, neurodegenerative disease, and neurobiology due to the achievements and advances in the knowledge and methodology in studying* Drosophila melanogaster*. The projection neurons (PNs) in antennal lobe of* Drosophila* transferring information from olfactory receptor neurons to mushroom body are known to be cholinergic, and PNs together with other interneurons in antennal lobe establish the complex neurocircuit processing the olfactory information. These provide an ideal model to investigate neurotransmission and neurofunction of cholinergic neurons which are fundamental excitative neurons in CNS of human related to memory [17].

In the present study, we investigated the effects of sevoflurane on neurotransmission and neurofunction after sevoflurane exposure and explored possible underlying mechanisms by using a* Drosophila* whole brain recording system [18].

## 2. Materials and Methods

### 2.1. Flies Stock

*Drosophila melanogaster* were reared in standard cornmeal agar medium accompanied with dry yeast at 24°C and 60% relative humidity. In order to reduce the errors, all experiments were performed using wild type canton-s flies 2-3 days before eclosion which was selected by red eyes and transparent wing in the puparium.

### 2.2. Sevoflurane Exposure Protocol

The sevoflurane groups (1% sevoflurane, exposure lasted 5 hours; 2% sevoflurane, exposure lasted 5 hours; 3% sevoflurane, exposure lasted 5 hours) were placed in a special anesthesia glass box, respectively. Gas was delivered through an anesthesia machine. Air was used as carrier and fresh air flow was controlled at 2 L/min. Gas was monitored by a monitor machine (Datex-Ohmeda, Louisville, KY, USA). Temperature in the experiment room was controlled at 24°C and humidity was 60%. 24 hours after sevoflurane exposure, the sevoflurane groups were subjected to experiment.

### 2.3. Electrophysiological Recording of PNs in Whole Brain Obtained from* Drosophila melanogaster*

*Drosophila* whole brains were obtained from control and postanesthesia flies 2-3 days before eclosion. The whole brains were dissected out of the head for experiment in external solution (101 mM NaCl, 1 mM CaCl_2_, 4 mM MgCl_2_, 3 mM KCl, 5 mM glucose, 1.25 mM NaH_2_PO_4_, 20.7 mM NaHCO_3_, pH 7.2, and Osm 250 mosM) additionally containing 20 units/mL papain with 1 mM l-cysteine. Pipettes (10–15 MΩ) pulled using a micropipette puller were targeted to PNs in the dorsal neuron cluster at the antennal lobe of brain which were mounted in an RC-26 perfusion chamber (Warner Instruments, Hamden, CT, USA) containing the external solution bubbled with 95% O_2_ and 5% CO_2_ (2 mL/min).

Whole-cell recordings were performed with pipettes filled with internal solution (102 mM K-gluconate, 0.085 mM CaCl_2_, 1.7 mM MgCl_2_, 17 mM NaCl, 0.94 mM EGTA, 8.5 mM HEPES, pH 7.2, and Osm 235 mosM). The internal solution for calcium current recording was similar except that K-gluconate was replaced by cesium gluconate. Whole-cell configuration was achieved in voltage-clamp mode after Giga ohm seals were completed. Slow and fast capacitance compensation was automatically completed. Access resistance was continuously monitored during the experiments. Current-clamp and voltage-clamp recordings were performed using patch-clamp system.

In order to record cholinergic mEPSCs, TTX (1 *μ*M) and picrotoxin (10 *μ*M) were added to the external solution to block voltage-gated sodium currents and GABAergic synaptic currents, respectively. While recording calcium current, TTX (1 *μ*M), TEA (10 mM), and 4-AP (1 mM) were added. All experiments were performed at room temperature and only one cell was recorded in each brain.

All electrophysiological experiments were performed through a BX51WI upright microscope (Olympus, Lehigh Valley, PA). Data, obtained by EPC10 amplifier (HEKA Elektronik, Lambrecht/Pfalz, Germany), were filtered at 5 kHz using a built-in filter and digitized at 5 kHz. Data analysis was performed by the pClamp10 Clampfit software (Molecular Devices). mEPSCs were analysed by Mini Analysis Software (Synaptosoft, Decatur, GA). Events were accepted for analysis only if they were asymmetrical with a rising phase faster than 3 ms and a more slowly decaying phase. In addition, the threshold criterion for inclusion was 3 pA.

### 2.4. Biocytin Staining and Immunohistochemistry Images

In order to identify and confirm that the cells recorded were PNs, cells recorded were stained by biocytin. While recording, cells were injected with biocytin through recording pipette filled with internal solution containing biocytin in whole-cell recording mode for at least 30 min. After recording, the brains were collected and fixed in 4% formaldehyde in PBS at 4°C for 10 h. Then brain was rinsed in 1% PBS three times, blocked, and incubated in a blocking buffer (0.1 M PBS, 0.1% Triton X-100, and 1% BSA) containing streptavidin-Cy3 (Molecular Devices) for 3 h at room temperature. After incubation, the brain was washed three times with 5 min intervals in PBS. A BX51WI microscope with a ×40 objective and confocal camera was used to acquire photos of dendritic arborization of the PNs in the antennal lobe.

### 2.5. Statistic Analysis

Data represented as mean ± S.D. Comparisons between groups were performed by analysis of one way ANOVA followed by Bonferroni-Dunn post hoc test or independent sample* t*-tests. And *P* < 0.05 was considered significant.

## 3. Results

### 3.1. Confocal Image of PNS of* Drosophila* Pupa Two Days before Eclosion

**Figure 1:**
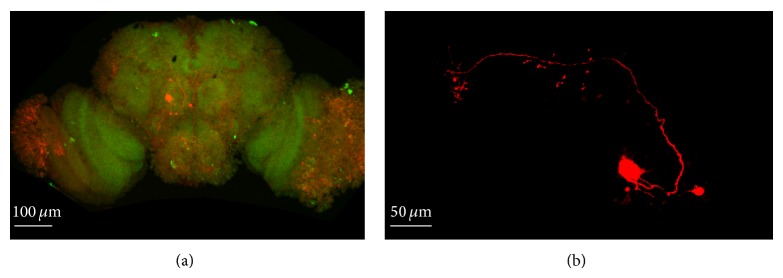
Confocal images showed the location and morphology of labelled PNs in* Drosophila* brain. (a) Confocal images of* Drosophila* pupa brain with biotin labelled olfactory PNs showed the detail morphology of the recorded neurons (Figure 1(a)). (b) Single neuron has been labeled (Figure 1(b)). There is one major branch of the soma stalk of the visual projection neuron, and this branch curves dorsomedially, giving off several small collaterals.

In Figure 1, the morphology of PNs was showed in the isolated brain. PNs are members of the synaptic net in which fast excitatory synaptic transmission was mediated by the *α*-bungarotoxin- (*α*-BTX-) sensitive nicotinic acetylcholine receptors (nAChRs). Previous studies have indicated that PNs receiving information from antennal lobe project their axon with cholinergic output to Kenyon cells in* Drosophila*. In the present study, In order to identify and confirm that the cells recorded were PNs all brains recorded were subjected to biocytin staining and immunohistochemistry imaging. Results obtained from cells which were in accordance with the morphology of PNs were accepted to be analyzed.

### 3.2. Property of Spontaneous Action Potentials (sAP) of* Drosophila* Pupa Two Days before Eclosion Was Not Altered after Sevoflurane Exposure

**Figure 2:**
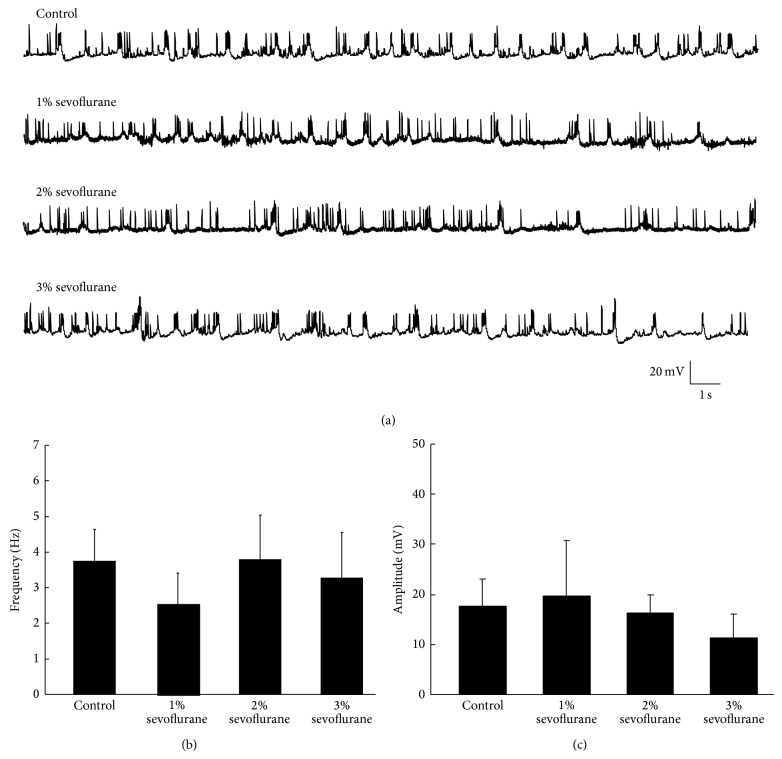
Effects of sevoflurane on the frequency and amplitude of sAP recorded from PNs in the* Drosophila* brain. In the current-clamp mode, sAP was recorded. (a) sAP traces recorded from PNs in control and sevoflurane group. (b) Frequency of sAP in each concentration of sevoflurane was not changed (*P* > 0.05, *n* = 6, *N* = 24). (c) Amplitude of sAP in each concentration of sevoflurane was not changed (*P* > 0.05, *n* = 6, *N* = 24).

In order to study whether sAP which is the fundamental property of neurofunction was changed 24 hours after sevoflurane exposure, we recorded the sAP of PNs from the whole brains isolated from CS pupa 2-3 days before eclosion of control and postanesthesia groups in whole-cell current clamp. While under the whole-cell current- clamp mode, sAP of PNs was recorded. The number of sAP which was over shooting more positive than 0 mV was counted; meanwhile peak amplitude was measured (Figure 2(a) *n* = 6, *N* = 24). Neither frequency nor amplitude was altered 24 hours after sevoflurane exposure when compared to control group (*P* > 0.05) (Figures 2(b) and 2(c)).

### 3.3. Cholinergic Miniature Excitatory Postsynaptic Currents (mEPSCs) of* Drosophila* Pupa Two Days before Eclosion Were Altered after Sevoflurane Exposure

**Figure 3:**
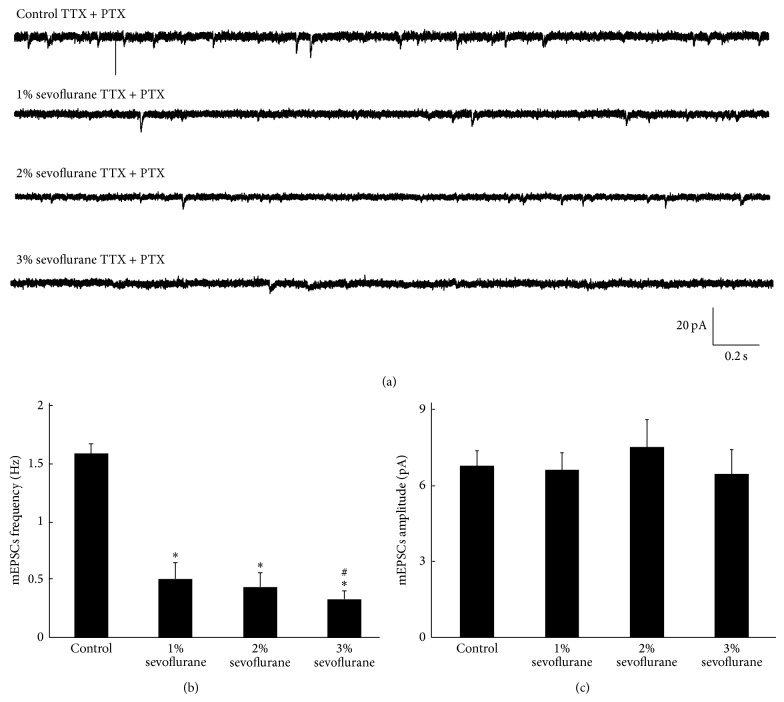
Cholinergic mEPSCs were altered 24 h after sevoflurane exposure. (a) While neurons were held on −70 mV in voltage-clamp mode, mEPSCs traces were recorded in each group in the presence of TTX and PTX. (b) 24 h after sevoflurane exposure, frequency of mEPSc was significantly reduced when compared to control (*P* < 0.05, *n* = 6, *N* = 24), (c) while the amplitude of mEPSCs was not changed (*P* > 0.05, *n* = 6, *N* = 24).

mEPSCs recorded from PNs have been proved to be mediated by acetylcholine by adding curare a nicotinic neuromuscular acetylcholine receptors antagonist. In the present study, mEPSCs were recorded from PNs in the presence of TTX and PTX in control and sevoflurane groups 24 hours after sevoflurane exposure. In control group, mean frequency and amplitude of mEPSCs were 1.60 ± 0.0875 and 6.80 ± 0.614 pA, respectively. In sevoflurane groups (1%, exposure lasted 5 hours; 2%, exposure lasted 5 hours; 3% exposure lasted 5 hours), mean frequency of mEPSCs was significantly reduced to 0.510 ± 0.140, 0.438 ± 0.134, and 0.329 ± 0.0765, respectively, when compared to control group (*P* < 0.05, *n* = 6, *N* = 24, Figure 3(b)) and it showed slight dose dependent effects (Figure 3(b)). However, amplitude of mEPSCs was not changed after sevoflurane exposure (*P* > 0.05, *n* = 6, *N* = 24, Figure 3(c)).

### 3.4. Calcium Currents of* Drosophila* Pupa Two Days before Eclosion Were Altered after Sevoflurane Exposure

**Figure 4:**
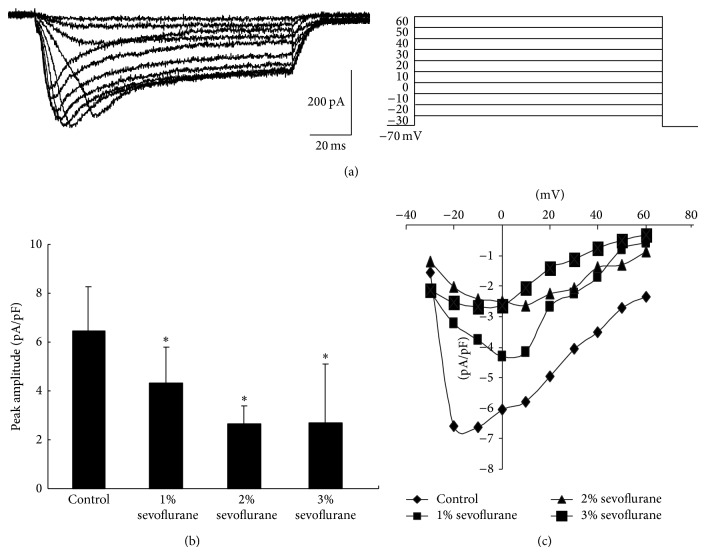
Calcium currents density was altered 24 h after sevoflurane exposure. (a) This showed the normal calcium currents traces which were recorded in the presence of TTX, TEA, and 4-AP, while recorded neurons were depolarized in a serial voltage step. (b) 24 h after sevoflurane exposure, peak amplitude of calcium currents density was significantly reduced when compared to control (*P* < 0.05, *n* = 6, *N* = 24). (c) Amplitude of calcium current density in each voltage step was shown in this graph. It is clear that peak amplitude of current density was reduced after sevoflurane exposure (*P* < 0.05, *n* = 6, *N* = 24).

In order to explorer the underlying mechanisms, we investigated whether sevoflurane would alter the calcium currents. In the experiment of recording calcium currents, internal solution which was the same as those used in recording mEPSCs except that potassium gluconate was replaced by cesium gluconate was used to record in the presence of TTX (1 *μ*M), TEA (10 mM), and 4-AP (1 mM). In order to reduce errors due to the size of the cells, data were showed in the form of current density. Data showed that mean peak amplitude in control group was 6.06 ± 1.65 pA/pF. And in sevoflurane groups (1%, exposure lasted 5 hours; 2%, exposure lasted 5 hours; 3% exposure lasted 5 hours), mean peak amplitude was significantly reduced to 4.32 ± 1.48 pA/pF, 2.65 ± 0.738 pA/pF, and 2.69 ± 2.40 pA/pF, respectively, when compared to control group (Figure 4).

## 4. Discussion

Presently, using* Drosophila *whose majority neurons are cholinergic whole brain recording system [18, 19], we are able to record cholinergic synaptic transmission in an intact nuerocircuit after anesthesia, which is hard to achieve in mammal animals.

In our study, all recorded neurons were showed in the brain through method of biocytin staining and immunohistochemistry. In order to reduce the error in experiments and make the data more reliable, data recorded from neurons in* Drosophila* brain whose location and morphology are in accordance with the PNs were accepted to further analysis (Figure 1).

In the present study, neither the frequency nor the amplitude of sAP were altered 24 hours after exposing* Drosophila* pupa to three concentrations of sevoflurane. It is well known that action potential is a fundamental property of neurons in mammal brain. It reflects ions that exchange across cell membrane through ion channel especially voltage-gate sodium channel. Previous studies suggested that sevoflurane while in clinical concentration inhibits sodium channel hence blocking action potential which is thought to lead to anesthesia effects [20]. All these indicated that fundamental property of neurons was not altered after sevoflurane exposure.

The mEPSCs are believed to result from spontaneous fusion of vesicles containing neurotransmitter to presynaptic terminal membrane. Frequency and amplitude of mEPSCs reflect the synaptic transmission activity. Alterations of frequency and amplitude of mEPSCs are ascribed to presynaptic and postsynaptic action, respectively [21]. Amplitude of mEPSCs reflects the response of postsynaptic receptor to a single vesicle while frequency reflects the possibility of vesicle fusion and release [22]. Previous studies indicated that the strength of synaptic transmission reflecting synaptic plasticity is dramatically modified by prior synaptic transmission activity, which can be expressed as metaplasticity. Studies from Sastry and Bhagavatula suggested that LTP is associated with an increase in frequency of mEPSCs [23]. Kushner et al. found that increasing phosphorylation of synapsin I in mice could enhance the density of docked vesicle in presynaptic membrane increasing the frequency of mEPSCs [24]. These changes facilitated subsequent induction of LTP and these mice showed an enhancement in learning and memory. Yang also found that melamine could reduce the frequency of mEPSCs hence diminishing the LTP and then impair the learning and memory of rats. It suggested that reducing synaptic transmission activity as shown in decreasing the frequency of mEPSCs could impair LTP [22, 25]. In order to determine whether synaptic transmission activity was modified, we measured the mEPSCs and found that the frequency of it was significantly reduced to 0.510 ± 0.140, 0.438 ± 0.134, and 0.329 ± 0.0765  24 h after sevoflurane exposure in sevoflurane groups, respectively, when compared to control group (1.6 ± 0.0875) while amplitude was not changed. Theses suggested that cholinergic synaptic transmission activity was inhibited 24 hours after sevoflurane exposure causing alteration of synaptic plasticity and subsequent learning and memory deficit through presynaptic mechanisms.

There are many factors that could contribute to modification of presynaptic transmitters release altering the frequency of mEPSCs. Studies showed that frequency of mEPSCs is associated with calcium. Lee et al. found that frequency of mEPSCs in cholinergic neurons was partially regulated by calcium currents [26]. Data showed that reducing influx of calcium through voltage-gate calcium channels decreased the frequency of mEPSCs, probability of neurotransmitter release from presynaptic membrane, by adding Co2+ which blocked calcium channels [27]. In order to explore the underlined mechanism that sevoflurane decreases the frequency of mEPSCs, calcium currents were measured 24 hours after sevoflurane exposure. And data showed that mean peak calcium currents density in sevoflurane groups was significantly reduced to 4.32 ± 1.48 pA/pF, 2.65 ± 0.738 pA/pF, and 2.69 ± 2.40 pA/p, respectively, 24 hours after sevoflurane exposure when compared to control group. It showed a slight dose dependent inhibition which was similar to that on the frequency of mEPSCs. This indicated that mean calcium currents were inhibited 24 hours after sevoflurane hence reducing the frequency of mEPSCs, which impaired the synaptic plasticity and subsequent learning and memory function. However, whether these effects would last for a life time and the detail mechanisms are not well understood and it needs to be elucidated in our further investigation.

## 5. Conclusion

In the present study, we found that, 24 hours after sevoflurane exposure, frequency of mEPSC and peak amplitude of calcium current density were reduced in slight dose dependent effects while amplitude of mEPSCs, frequency, and amplitude of sAP were not altered using* Drosophila melanogaster* whole brain recording model. These data indicated that sevoflurane may alter central nervous system synaptic plasticity partially through inhibiting calcium current 24 hours after sevoflurane exposure which decreased the presynaptic neurotransmitter release.
